# Weather Impact on Acute Myocardial Infarction Hospital Admissions With a New Model for Prediction: A Nationwide Study

**DOI:** 10.3389/fcvm.2021.725419

**Published:** 2021-12-14

**Authors:** Chen-Yu Li, Po-Jui Wu, Chi-Jen Chang, Chien-Ho Lee, Wen-Jung Chung, Tien-Yu Chen, Chien-Hao Tseng, Chia-Chen Wu, Cheng-I Cheng

**Affiliations:** ^1^Department of Finance, National Taichung University of Science and Technology, Taichung, Taiwan; ^2^Division of Cardiology, Department of Internal Medicine, Kaohsiung Chang Gung Memorial Hospital, Kaohsiung, Taiwan; ^3^Graduate Institute of Clinical Medicine Sciences, Chang Gung University, Taoyuan, Taiwan; ^4^Research Services Center for Health Information, Chang Gung University, Taoyuan, Taiwan; ^5^Clinical Informatics and Medical Statistics Research Center, Chang Gung University, Taoyuan, Taiwan; ^6^Department of Obstetrics and Gynecology, Chang Gung Memorial Hospital, Taoyuan, Taiwan; ^7^Gynecologic Cancer Research Center, Chang Gung Memorial Hospital, Taoyuan, Taiwan; ^8^School of Medicine, College of Medicine, Chang Gung University, Taoyuan, Taiwan

**Keywords:** acute myocardial infarction, weather, prediction, nationwide, generalized additive model

## Abstract

**Introduction:** Cardiovascular disease is one of the leading causes of mortality worldwide. Acute myocardial infarction (AMI) is associated with weather change. The study aimed to investigate if weather change was among the risk factors of coronary artery disease to influence AMI occurrence in Taiwan and to generate a model to predict the probabilities of AMI in specific weather and clinical conditions.

**Method:** This observational study utilized the National Health Insurance Research Database and daily weather reports from Taiwan Central Weather Bureau to evaluate the discharge records of patients diagnosed with AMI from various hospitals in Taiwan between January 1, 2008 and December 31, 2011. Generalized additive models (GAMs) were used to estimate the effective parameters on the trend of the AMI incidence rate with respect to the weather and health factors in the time-series data and to build a model for predicting AMI probabilities.

**Results:** A total of 40,328 discharges were listed. The minimum temperature, maximum wind speed, and antiplatelet therapy were negatively related to the daily AMI incidence; however, a drop of 1° when the air temperature was below 15°C was associated with an increase of 1.6% of AMI incidence. By using the meaningful parameters including medical and weather factors, an estimated GAM was built. The model showed an adequate correlation in both internal and external validation.

**Conclusion:** An increase in AMI occurrence in colder weather has been evidenced in the study, but the influence of wind speed remains uncertain. Our analysis demonstrated that the novel GAM model can predict daily onset rates of AMI in specific weather conditions.

## Introduction

Cardiovascular disease is one of the major causes of mortality globally. Cardiovascular diseases have been the leading cause of mortality in Taiwan since 2007, with only one step behind cancers. Acute myocardial infarction (AMI) plays an important role in cardiovascular death. The exact trigger of AMI may not be clearly defined, but previous epidemiological studies have identified some risk factors that might lead to the onset of AMI. These risk factors include heavy exercise and/or physical exertion, sexual activity, cocaine or marijuana abuse, and emotional stress. Over the past few decades, growing pieces of evidence have led to concerns about the potential effects of the environment on cardiovascular health. For instance, premature mortality was first highlighted to be attributed to environmental factors, such as air pollution in the investigation of the Great Smog of London in 1952 (1). In addition, individuals exposed to extreme weather temperature drop are highly prone to acquiring cardiovascular diseases was first reported by the Eurowinter Group in 1997 (2). Moreover, meteorological factors were estimated to demonstrate an attributable effect on the morbidity of myocardial infarction (MI) in a longitudinal population study in 1999 (3). Besides, Medina-Ramón M et al. stated that cardiovascular deaths were increased in patients exposed to temperature extremes in 2006 (4). In 2009, a systematic review identified 19 studies and reported a short-term effect of temperature on a daily timescale in 14 studies and a long-term effect in the rest of the studies. Overall, a significant short-term increased risk of MI at lower temperatures was revealed in 8 of 12 studies that included data relevant from the winter reports. Meanwhile, an increased risk of MI at higher temperatures was also seen in 7 of 13 studies. Thus, both hot and cold weather conditions had detrimental effects on the short-term risk of MI (5). Due to geographic variation and weather change, the prevalence of cardiovascular-related diseases has a statistically significant relationship with the incidence of AMI. However, prior pieces of research have focused exclusively on the effect of weather on AMI, especially in the northern higher latitude area (6–10). Except for the environmental factors, the underlying clinical conditions of the patients such as coronary artery disease (CAD) and other co-morbidities are also relevant to the onset of AMI. However, the attribution of AMI to the parameters of weather change, the main medications of cardiovascular diseases, and the risk factors of CAD are rarely explored. Hence, the study aimed to investigate if weather change was among the risk factors of coronary artery disease to influence AMI occurrence in Taiwan and to generate a model to predict the probabilities of AMI in specific weather and clinical conditions.

## Method

### Data Source and Study Design

Previously known as the “Bureau of National Health Insurance,” the National Health Insurance Administration (NHIA) is defined as a “financial and insurance public enterprise,” which is responsible for managing health insurance affairs, medical quality, research and development, manpower training, and information on the health care system and has provided the whole residents in Taiwan with an effective healthcare system since March 1995. Approximately 96% of the entire Taiwanese population have been registered in the health insurance program. Meanwhile, the National Health Insurance Research Database (NHIRD) had included all the medical details and reimbursement data for more than 97% of the hospitals and private clinics around the island since 1996, indicating the medical information in the NHIRD was enormous and sufficient to represent the history of various diseases for the Taiwanese population.

This study mainly used two data sets, namely, the healthcare data from the NHIRD and the weather data from the Central Weather Bureau (CWB), to investigate the relationship between common risk factors and weather changes in the occurrence of AMI (ICD-9-CM code: 410; International Classification of Diseases, Ninth Revision, Clinical Modification). This study focused on those patients who visited the emergency department (ED) and were admitted between January 1, 2008 and December 31, 2011. The inclusion criterion of the study was those patients who were diagnosed with AMI, and the exclusion criteria were as follows: (1) those who did not visit the ED; (2) those who visited the hospital that is not located on the main island of Taiwan.

Additionally, the possible risk factors of AMI in the medical histories were also extracted from the NHIRD. To avoid the influence of potential coding errors, risk factors and co-morbid diseases such as diabetes mellitus, hypertension, stroke, etc., were extracted from (1) discharge diagnoses of hospitalization for this index AMI event and any other cause prior to this AMI event or (2) outpatient diagnosis if corresponding medications were prescribed. These risk factors for analyses included age, gender, thyroid disorder (ICD-9-CM: 193, 2409, 2429, 2449, 64813), fibrinolysis (ICD-9-CM: 2866), percutaneous coronary intervention (PCI; operation code: 360), coronary artery bypass grafting (CABG; operation code: 361–369, v4581), heart failure (HF; ICD-9-CM: 4280, 4281), stroke (ICD-9-CM: 430–437), malignant dysrhythmia (ICD-9-CM: 4260, 42612, 42613, 42651, 42652, 42654, 4271, 4274, 4275), cardiogenic embolism (ICD-9-CM: 78551), atrial fibrillation (AF; ICD-9-CM: 42731), hypertension (ICD-9-CM: 401–405, 4372, 36211), diabetes mellitus (DM; ICD-9-CM: 250, 3572, 36201, 36202, 36641), hyperlipidemia (ICD-9-CM: 272), chronic obstructive pulmonary disease (COPD; ICD-9-CM: 490–496), chronic kidney disease (CKD; ICD-9-CM: 585), dialysis (ICD-9-CM: 3995, 5498), CAD (ICD-9-CM: 414), peripheral arterial disease (ICD-9-CM: 4439), revascularization (ICD-9-CM: 360–369, v4581), respiratory failure (ICD-9-CM: 51881), major bleeding (ICD-9-CM: 4560, 4590, 5780, 5781, 5789, 5693, 531, 532, 534, 535, 562, 569, 5997, 7863), pulmonary embolism and deep vein thrombosis (ICD-9-CM: 4151, 453, 673), and acute kidney injury (ICD-9-CM: 584). Medications including antiplatelet agents (aspirin or clopidogrel or both) and statin were extracted from outpatient prescriptions within 3 months prior to this ED visit for AMI by using ATC codes as following: clopidogrel (B01AC04), aspirin (B01AC06), simvastatin (C10AA01), lovastatin (C10AA02), pravastatin (C10AA03), fluvastatin (C10AA04), atorvastatin (C10AA05), rosuvastatin (C10AA07), pitavastatin (C10AA08), and ezetimibe/simvastatin (C10BA02).

Weather data were used for those collected one day before the index day of AMI. Daily weather data included air temperature, air humidity, rainfall, sunshine duration, and wind speed from 20 of 25 weather stations, which were systematically located within inhabitant-intensive areas around Taiwan. Thirteen results of weather variables were regarded as minimum, maximum, or average per day. Change in air temperature was measured as the difference between the maximum and minimum air temperature.

### Statistical Analysis

This study was performed mainly to investigate the effects of weather conditions and the clinical factors on the daily onset rate of AMI in 2008–2011. The clinical factors were analyzed by univariate logistic regression. The statistically significant factors will be chosen for further evaluation. *P* < 0.05 was considered statistically significant. Considering that these weather data were greatly complex, the generalized additive model (GAM) (11), which is a nonparametric regression approach, provides powerful tools that enable relatively multiple linear and non-linear relationships to be explored in the time-series data simultaneously. The weather factors were analyzed by GAM. To estimate the effective parameters on the trend of the AMI incidence rate with respect to the weather and health factors in the time-series data, we adopted the GAM to explore nonparametrically how factors affected a response variable (12, 13). Factors with statistical significance in GAM were then put in an equation to estimate the daily incidence of AMI. *P* < 0.05 was considered statistically significant. We used internal validation and external validation to evaluate the relationship between the estimated incidence of AMI and the true incidence of AMI.

Continuous variables are presented as mean (*SD*). Categorical variables are displayed as counts and percentages. Furthermore, Student's *t*-test was used, and *P*-values for trend were calculated as the baseline characteristics of patients with AMI in 2008–2010. The logistic regression for the time-series model was used to estimate the hazard ratio of risk factors associated with AMI in the study period. To determine the quality statistical model relative to each of the other models, we adopted the Akaike Information Criterion (AIC) and the adjusted R-squared. All analyses were conducted using the SAS statistical software, version 9.4 (SAS Institute Inc., Cary, North Carolina, USA).

## Results

**Figure 1:**
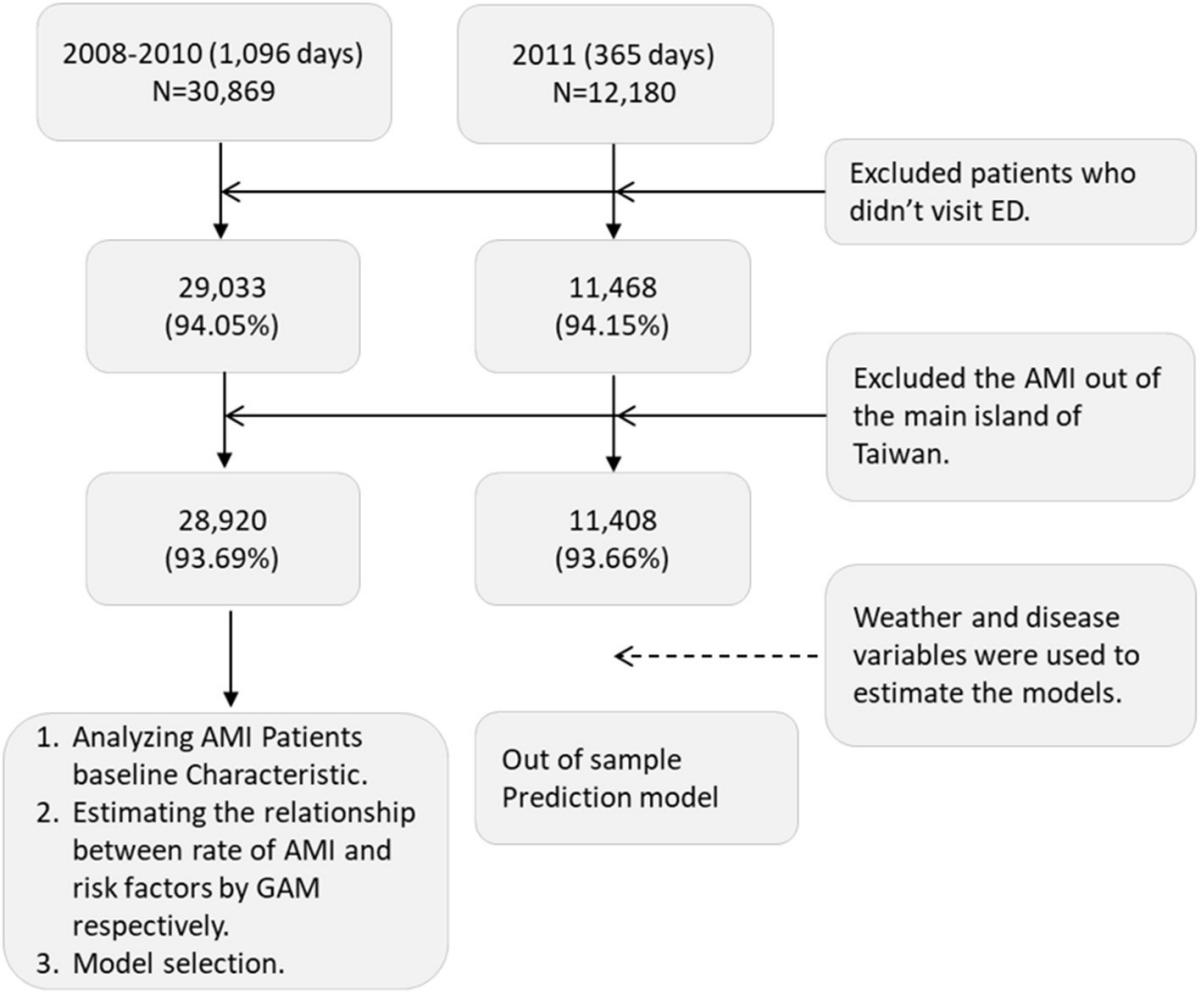
Algorithm of selection of AMI patient from NHIRD. This figure showed the initial number from database and the number after exclusion. The exclusion criteria were listed in the right two boxes. N, number of AMI admissions; ED, emergency department; AMI, acute myocardial infarction; GAM, generalized additive model.

**Figure 2:**
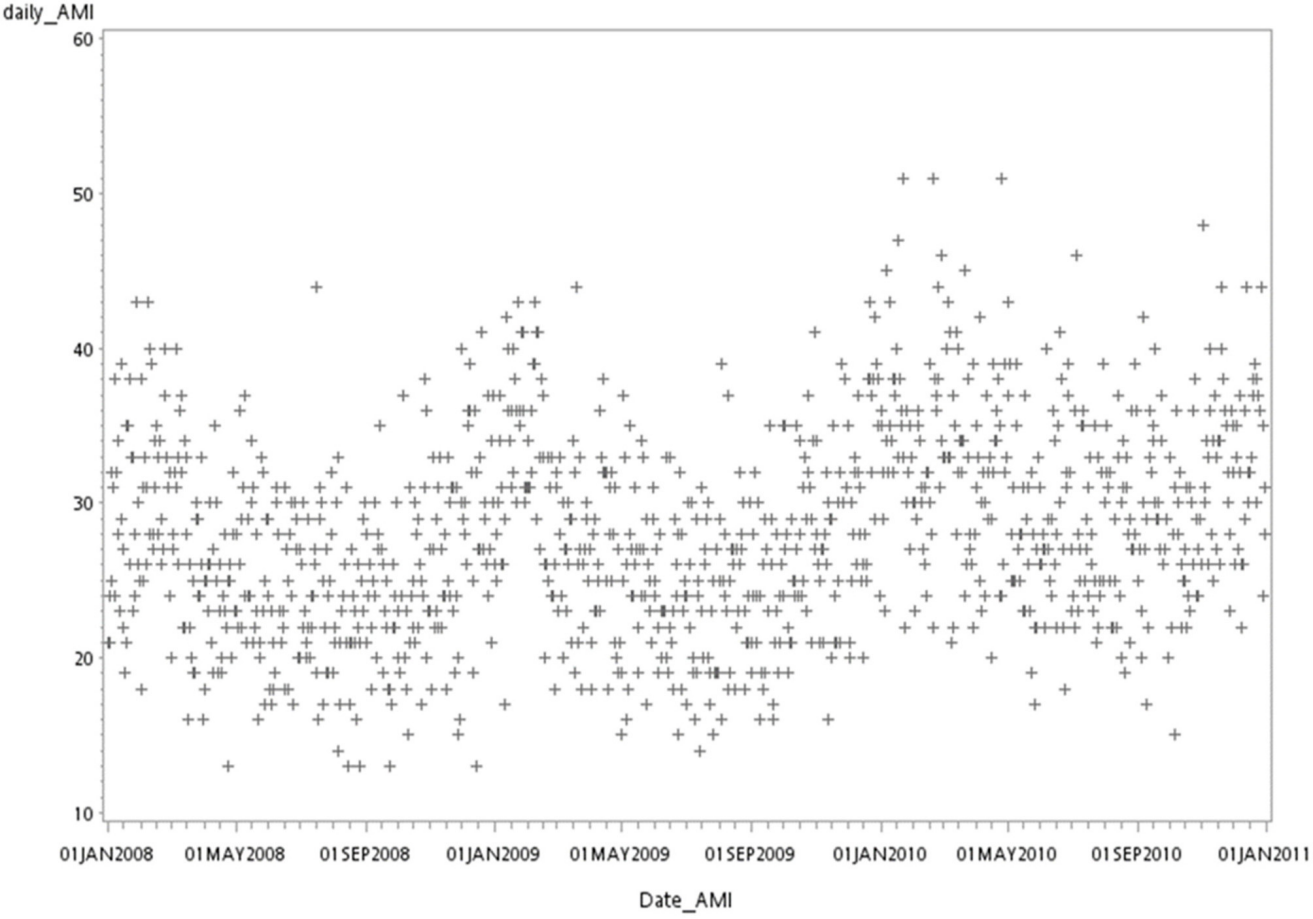
Actual AMI number in Taiwan from 2008 to 2010. This figure showed the number of AMI admissions from 2008 to 2010. Each marker represents the number of AMI admissions in 1 day.

During the 4 study years, 43,049 patients were recorded to have AMI (ICD-9-CM code: 410) in the NHIRD. After exclusion, 40,328 (29,055 men, 72%) patients remained for further analyses (Figure 1). The number (from 9,005 to 11,408 per year) and the mean age (from 66 to 66.5 years) of the patients with AMI increased annually, especially for those of age over 85 years (from 8 to 10.5%). The incidences of AF (from 3.5 to 3.7%) and CKD (from 6.8 to 9.8%) had increased significantly as well. However, CABG, CAD, cardiogenic embolism, PCI, and revascularization had an inverse trend against time (Table 1). The daily incidence rate of AMI had a clear cycle, in which it reached a peak in winter and went down to the trough in summer during 2008–2010 (Figure 2).

**Table 1:** Baseline characteristics of acute myocardial infarction (AMI) patients.

**Year**	**2008**	**2009**	**2010**	**2011**	**P trend**
Patient number	9,005	9,288	10,627	11,480	
Male–No. (%)	6,503 (72.2)	6,727 (72.4)	7,594 (72.1)	8,231 (72.1)	0.2748
Age–year	66.0 ± 14.7	66.0 ± 14.9	66.4 ± 14.9	66.5 ± 15.0	0.0673
≤ 64 years–No. (%)	4,069 (45.2)	4,206 (45.3)	4,831 (45.5)	5,232 (45.9)	0.0244
65–74 years–No. (%)	1,996 (22.2)	2,046 (22.0)	2,208 (20.8)	2,284 (20.0)	0.0196
≥75 years–No. (%)	2,940 (32.6)	3,036 (32.7)	3,588 (33.8)	3,892 (34.1)	0.0293
Medical history–No. (%)					
Myocardial infarction	1,013 (11.2)	982 (10.6)	1,034 (9.7)	825 (7.2)	0.0536
Thyroid disease	92 (1.0)	98 (1.1)	97 (0.9)	113 (1.0)	0.4583
Fibrinolysis	3 (0.03)	1 (0.01)	1 (0.01)	2 (0.02)	0.4066
Percutaneous coronary intervention	448 (5.0)	426 (4.6)	424 (4.0)	303 (2.6)	0.0401
Coronary artery bypass grafting	47 (0.5)	31 (0.3)	30 (0.3)	12 (0.1)	0.0196
Heart failure	976 (10.8)	933 (10.0)	1,113 (10.5)	1,154 (10.1)	0.3457
Stroke *	898 (10.0)	995 (10.7)	1,142 (10.7)	1,206 (10.5)	0.4108
Malignant arrhythmia	94 (1.0)	94 (1.0)	78 (0.7)	91 (0.8)	0.1421
Cardiogenic embolism	64 (0.7)	52 (0.6)	51 (0.5)	43 (0.4)	0.0083
Atrial fibrillation	312 (3.5)	326 (3.5)	385 (3.6)	429 (3.7)	0.0160
Hypertension	4,332 (48.1)	4,481 (48.2)	5,319 (50.1)	5,584 (48.6)	0.5054
Diabetes mellitus	2,587 (28.7)	2,702 (29.1)	3,212 (30.2)	3,433 (29.9)	0.1347
Hyperlipidemia	1,789 (19.9)	1,890 (20.3)	2,325 (21.9)	2,270 (19.8)	0.8343
Chronic obstructive pulmonary disease	1,168 (13.0)	1,086 (11.7)	1,260 (11.9)	1,377 (12.0)	0.3792
Chronic kidney disease	615 (6.8)	713 (7.7)	949 (8.9)	1,127 (9.8)	0.0029
Dialysis	172 (1.9)	184 (2.0)	270 (2.5)	280 (2.4)	0.1296
Coronary artery disease	2,330 (25.9)	2,303 (24.8)	2,446 (23.0)	2,533 (22.1)	0.0068
Peripheral artery disease	93 (1.0)	90 (1.0)	105 (1.0)	110 (1.0)	0.1960
Revascularization	487 (5.4)	447 (4.8)	447 (4.2)	315 (2.7)	0.0289
Respiratory failure	224 (2.5)	236 (2.5)	306 (2.9)	261 (2.3)	0.8443
Major bleeding **	520 (5.8)	567 (6.1)	658 (6.2)	631 (5.5)	0.6976
Gastrointestinal bleeding	173 (1.9)	176 (1.9)	209 (2.0)	201 (1.8)	0.3934
Venous thromboembolism	43 (0.5)	39 (0.4)	56 (0.5)	59 (0.5)	0.4203
Acute kidney injury	97 (1.1)	133 (1.4)	173 (1.6)	196 (1.7)	0.0413

*Stroke included intracranial hemorrhage. **Major bleeding included GI bleeding, hematuria, hemoptysis, retinal hemorrhage.

Possible risk factors for AMI were shown in Table 2, which included age, male sex, and comorbidities as heart failure, hypertension, DM, dyslipidemia, COPD, and CKD (dialysis). The time-series odds ratio for the onset of AMI was significantly higher in the patients who possessed these risk factors. Factors such as prior MI, AF, statin therapy, and antiplatelet therapy were then found to be negatively correlated to the onset of AMI in univariate logistic regression.

**Table 2:** Univariate logistic regression of odds ratio of demographics for AMI event.

**Risk factor**	**Odds Ratio**	**95% of Confidence**	***P* value**
		**Interval**	
Age	1.046	(1.046, 1.046)	<0.0001
Male	2.475	(2.471, 2.480)	<0.0001
Myocardial infarction	0.792	(0.694, 0.851)	<0.0001
Heart failure	1.070	(1.026, 1.114)	0.0013
Stroke	1.027	(0.990, 1.065)	0.1534
Atrial fibrillation	0.810	(0.763, 0.860)	<0.0001
Hypertension	1.198	(1.182, 1.214)	<0.0001
Diabetes mellitus	1.484	(1.457, 1.511)	<0.0001
Hyperlipidemia	1.199	(1.176, 1.222)	<0.0001
COPD*	1.840	(1.774, 1.908)	<0.0001
CKD** or dialysis	1.179	(1.120, 1.240)	<0.0001
Use of statin	0.090	(0.084, 0.098)	<0.0001
Use of antiplatelet agent***	0.092	(0.090, 0.094)	<0.0001

*COPD: chronic obstructive pulmonary disease; **CKD: chronic kidney disease; ***Antiplatelet agent: aspirin or clopidogrel.

**Figure 3:**
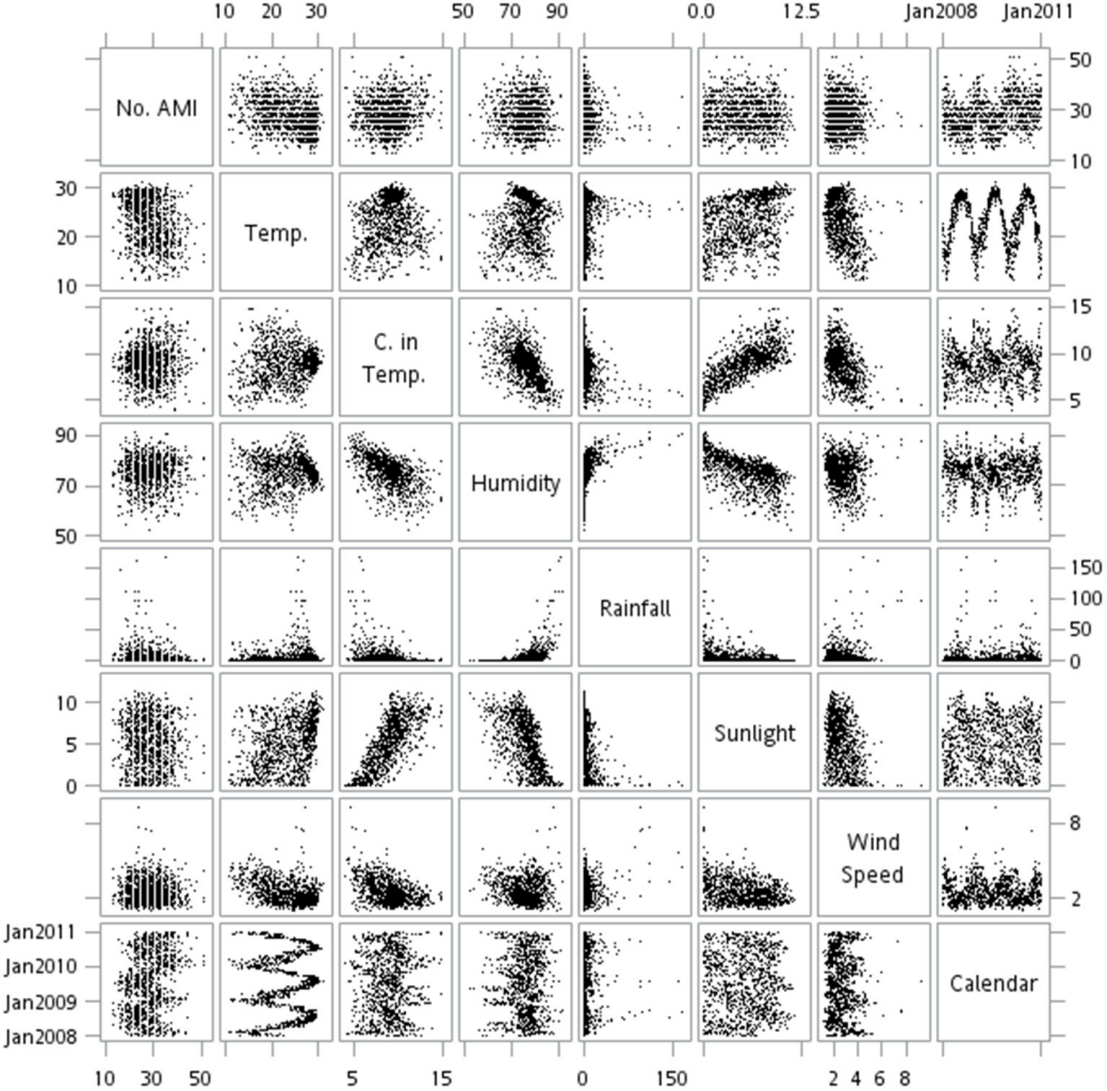
Matrix plots of the relationships between AMI daily events and weather factors in Taiwan from 2008 to 2010. The multiple scatter plots present the relationship between the daily rate of AMI admissions and the weather factors in 2008–2010. Most relationship were non-linear in this figure. No. AMI, number of acute myocardial infarction per day; Temp., the average of temperature (oC) per day; C. in Temp., change in temperature (oC) within 1 day; Humidity: the average of humidity per day; Rainfall: the amount of rainfall (millimeter) per day; Sunlight: the average of sunlight (hours); Wind Speed: the average of wind speed (meter per second) per day.

Weather change, such as low/high air temperature, change in air temperature, humidity, rainfall, sunshine duration, and wind speed, affects the occurrence of AMI in this study. The relationship between the daily occurrence rate of AMI and the weather of the study was shown in Figure 3, indicating the relationship was either slightly linear or nonlinear. However, only four factors were found to demonstrate significant roles in the causation of AMI onset in the first GAM approach, which were minimum temperature, change in air temperature, sunshine duration, and maximum wind speed (Tables 3-1, 3-2).

**Table 3-1:** GAM estimating the significant linear association of weather factors and AMI admission.

		**Parameter estimate**	**Standard error**	***P* value**
**Linear**	Intercept	0.1589	0.0240	<0.0001
	Minimum temperature	−0.0012	0.0003	0.0004
	Temperature difference	0.0015	0.0008	0.0940
	Humidity	0.0002	0.0002	0.3592
	Rainfall amount	0.0001	0.0001	0.2592
	Sunshine time	−0.0004	0.0006	0.4929
	Maximum wind speed	−0.0006	0.0004	0.0930

**Table 3-2:** GAM estimating the significant non-linear association of weather factors and AMI admission.

		**Sum of squares**	**Chi-square**	***P* value**
**Non-linear**	Minimum temperature	0.0028	2.9215	0.4039
	Temperature difference	0.0012	1.3062	0.7276
	Humidity	0.0025	2.5724	0.4624
	Rainfall amount	0.0050	5.1053	0.2039
	Sunshine time	0.0129	13.101	0.0044
	Maximum wind speed	0.0056	5.7650	0.1235

Tables 4-1, 4-2 illustrate that the combination of these 17 statistically significant variables, which involve weather and risk factors, was associated with the daily rate of AMI occurrence by the GAM approach. Because one of the statistically significant factors, particularly myocardial infarction, had conflict in the GAM and equation, we removed myocardial infarction and tried analyzing stroke in GAM. The results of the daily onset rate of AMI were shown in Tables 4-1, 4-2. In Table 4-1, linear effects of the daily AMI onset rate were found to be positively related to heart failure and statin therapy prior to MI but negatively related to minimum temperature, maximum wind speed, and antiplatelet therapy. Besides, non-linear effects of the daily AMI onset rate were found to be significantly associated with age, male sex, and sunshine time. Stroke still showed statistically insignificance in both linear and non-linear effects. The statistically significant factors were put in the equation estimating the onset of AMI for a subject that carries certain risk factors in a specified weather condition.

**Table 4-1:** Linear effect of weather factors and demographics in GAM.

	**Parameter**	**Standard**	***t* value**	***P*-value**
	**Estimate**	**Error**		
Intercept	0.1853	0.0288	6.43	<0.0001
Age	4.87 × 10^−4^	3.41 × 10^−4^	1.43	0.1544
Male	2.17 × 10^−3^	1.16 × 10^−2^	0.02	0.9852
Minimum temperature^#^	−2.31 × 10^−3^	2.88 × 10^−4^	−8.01	<0.0001
Change in Temperature^#^	9.98 × 10^−4^	8.60 × 10^−4^	1.16	0.2459
Gust peak speed^#^	−6.31 × 10^−4^	3.01 × 10^−4^	−2.09	0.0364
Sunshine time^#^	−5.56 × 10^−4^	5.41 × 10^−4^	−1.03	0.3042
Heart failure^#^	3.54 × 10^−4^	1.09 × 10^−4^	3.24	0.0012
Stroke^#^	−5.94 × 10^−5^	3.75 × 10^−5^	−1.59	0.1130
Atrial fibrillation^#^	2.02 × 10^−4^	1.41 × 10^−4^	1.43	0.1518
Hypertension^#^	9.62 × 10^−6^	1.40 × 10^−5^	0.68	0.4948
Diabetes mellitus^#^	−2.00 × 10^−5^	2.61 × 10^−5^	−0.77	0.4437
Hyperlipidemia^#^	−5.18 × 10^−5^	3.01 × 10^−5^	−1.72	0.0861
COPD*^,^^#^	1.05 × 10^−5^	3.30 × 10^−5^	0.32	0.7499
CKD**^,^^#^	−8.97 × 10^−7^	4.07 × 10^−6^	−0.22	0.8256
Use of antiplatelet agent***,^#^	−1.07 × 10^−4^	2.32 × 10^−5^	−4.62	<0.0001
Use of statin^#^	1.49 × 10^−4^	3.33 × 10^−5^	4.48	<0.0001

*COPD: chronic obstructive pulmonary disease; **CKD: chronic kidney disease; ***Antiplatelet agent: aspirin or clopidogrel; ^#^: denote the outcome of a variable at the day before.

**Table 4-2:** Non-linear effect of weather factors and demographics in GAM.

	**Degree of**	**Sum of**	**Chi-square**	***P*-Value**
	**freedom**	**squares**		
Age	3.000	0.0104	9.8729	0.0197
Male	3.000	0.0165	16.1837	0.0010
Minimum temperature^#^	3.000	0.0021	2.0751	0.5570
Change in Temperature^#^	3.000	0.0018	1.8229	0.6100
Gust peak speed^#^	3.005	0.0044	4.4100	0.2212
Sunshine time^#^	3.000	0.0135	13.3556	0.0039
Heart failure^#^	3.000	0.0054	5.4143	0.1439
Stroke^#^	3.000	0.0009	0.9618	0.8105
Atrial fibrillation^#^	3.000	0.0059	5.8755	0.1178
Hypertension^#^	3.000	0.0014	1.3995	0.7057
Diabetes mellitus^#^	3.000	0.0009	0.9241	0.8196
Hyperlipidemia^#^	3.000	0.0019	1.9508	0.5827
COPD*^, #^	3.000	0.0006	0.5711	0.9030
CKD**^, #^	3.000	0.0041	4.0029	0.2611
Use of antiplatelet agent***^, #^	3.000	0.0016	1.5918	0.6612
Use of statin	3.000	0.0025	2.4444	0.4854

*COPD: chronic obstructive pulmonary disease; **CKD: chronic kidney disease; ***Antiplatelet agent: aspirin or clopidogrel; ^#^: denote the outcome of a variable at the day before.

From the results in Tables 4-1, 4-2, several coefficients with statistical significance were chosen to estimate the incidence of AMI on a specific day and are expressed as follows (Equation 1):


$$\begin{array}{l} {ŷ}_{t}=0.19770\;+\;0.000453x_{1}+0.00170x_{2}-0.00277x_{\left(3,t-1\right)}\\ \;-0.000675x_{\left(4,t-1\right)}-0.000107x_{\left(5,t-1\right)}+0.000336x_{\left(6,t-1\right)}\\ \;-0.000130x_{\left(7,t-1\right)}+0.0000941x_{\left(8,t-1\right)}+\overset{8}{\underset{\left(i=1\right)}{\sum }}\left[{Ŝ}_{i}\left(x_{i}\right)\right] \end{array}$$

where ŷ_*t*_ denoted the expected value of the daily AMI onset rate (per 100,000 people) among people above 18 years old at the day *t*, *x*_1_ denoted the average age (years), *x*_2_ denoted the sex (male = 1, female = 0), *x*_3, *t*−1_ denoted the minimum temperature (°C) at the day *t*−1, *x*_4, *t*−1_ denoted the maximum wind speed (m/s), *x*_5, *t*−1_ denoted the sunshine duration (hours), *x*_6, *t*−1_ denoted the number of visits for HF, *x*_7, *t*−1_ denoted the number of patients using antiplatelet agent, and *x*_8, *t*−1_ denoted the number of patients using a statin. In addition, Ŝ_*i*_(*x*_*i*_) denoted a linear-adjusted nonparametric estimates smooth function of x, i for i =1, 2, 3, …, and 8.

**Figure 4:**
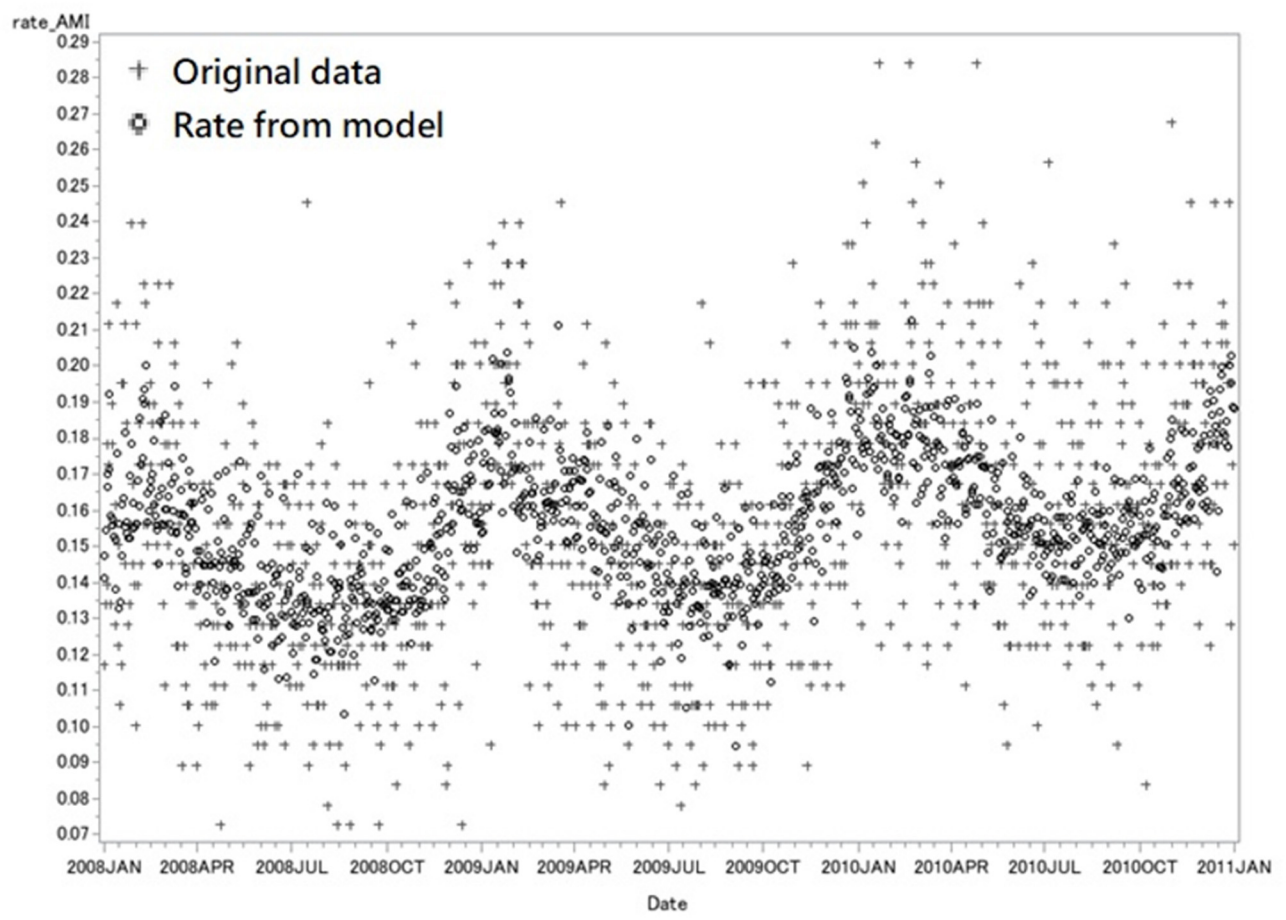
Internal validation with data from 2008 to 2010. This figure showed the original rate of daily AMI admission (cross) and the predicted rate from model (circle) from 2008 to 2010. Each marker represents the rate of AMI in 1 day. The rate of AMI is the number of AMI admission per 100,000 population in one single day. The AIC of internal validation was −4421.195, and the explained deviance was 25.4%.

For internal validation, we used the reduced regression model, considering that it yielded better performance in AIC than the full regression model. Under the null hypothesis, the two models were the same, with one having an insignificant *P*-value of 0.6991. The plot of the AMI rate with respect to the weather and risk factors was depicted based on Equation (1) between 2008 and 2010, and the cross/circle points are represented as the raw/estimated rate of AMI, respectively (Figure 4). Shown in Figure 4, a cycle was found for the estimated onset rate of AMI, in which the lowest point (0.144 ± 0.007 per 100,000 population) was noticed in summer (May–October) and the highest (0.176 ± 0.011 per 100,000 population) in winter (December–February). The AIC of internal validation was −4421.195, and the explained deviance was 25.4%. Furthermore, a lower temperature was associated with a higher onset rate of AMI when the temperature was below 18°C, and a drop of 1° when the air temperature was below 15°C was associated with a relative increase of 1.6% of AMI incidence. For example, the onset rate of AMI in temperature 14°C was increased by 1.016 times compared to the onset rate of AMI in temperature 15°C. The onset rate of AMI increased by 0.184 ± 0.013 per 100,000 population as the temperature decreased to <10°C. Moreover, the AMI rate increased approximately 0.3–1.2% yearly.

**Figure 5:**
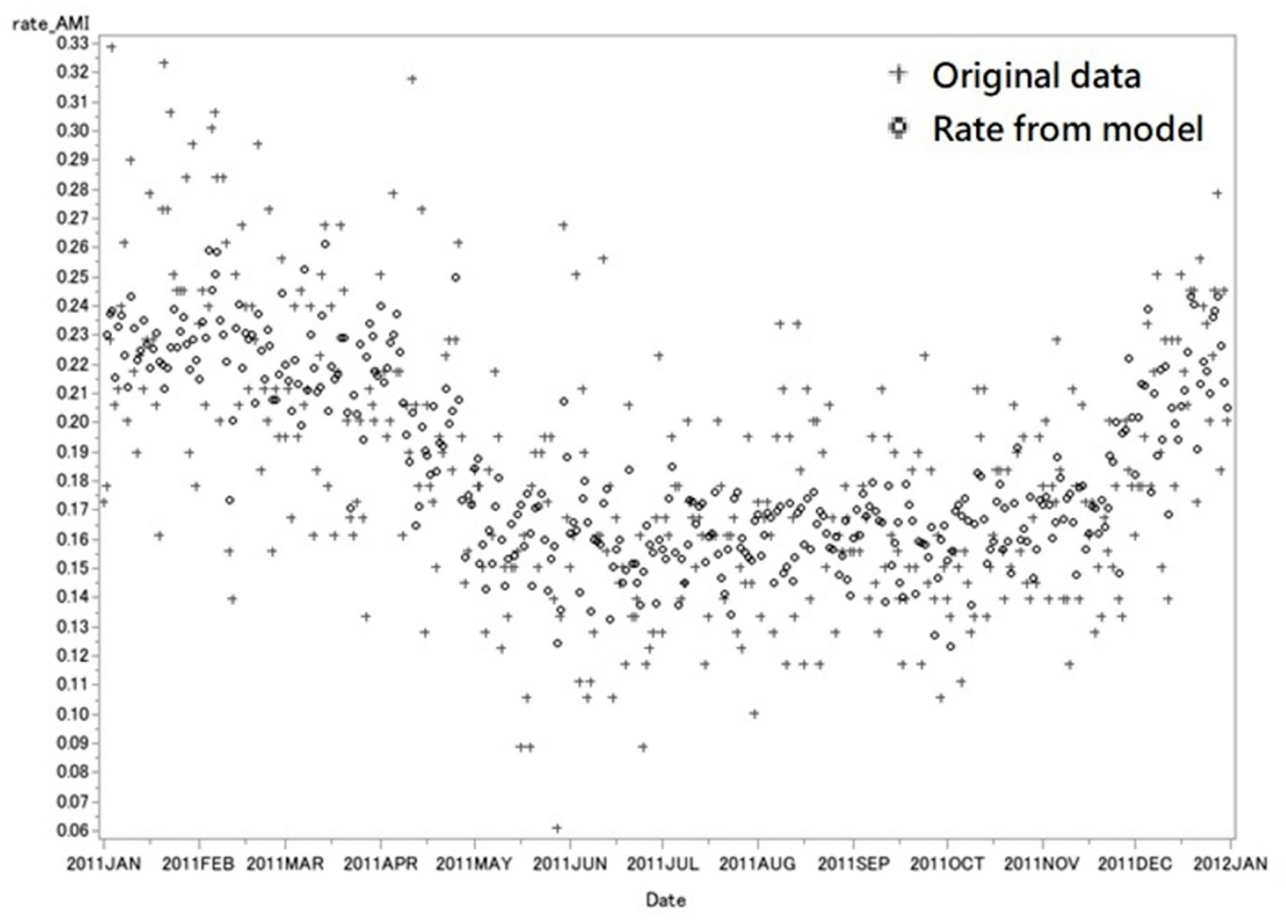
External Validation with data in 2011. This figure showed the original rate of daily AMI admission (cross) and the predicted rate from model (circle) in 2011. Each marker represents the rate of AMI in 1 day. The rate of AMI is the number of AMI admission per 100,000 population in one single day. The AIC of internal validation was −1400.293, and the explained deviance was 47.3%.

Similar results were obtained and shown in Figure 5 when some other data collected as of the same period in 2011 as the study data were analyzed using Equation (1) to test the practicability of the model, which as well demonstrated the peak of the onset rate of AMI (0.221 ± 0.017 per 100,000 population) in winter and the bottom (0.161 ± 0.013 per 100,000 population) in summer. The AIC of internal validation was −1400.293, and the explained deviance was 47.3%.

## Discussion

Our study revealed that the daily onset rate of AMI was significantly associated with weather. To our knowledge, this study is by far the largest one to analyze the association of AMI incidences with environmental factors and the prediction model we design is the first to integrate both weather and cardiovascular risk factors to evaluate the onset of AMI. After analyses, the onset rate of AMI was found highest in the winter and lowest in the summer. Lower minimum temperature and maximum wind speed were significantly related to a higher AMI rate. When it got cooler as the air temperatures were below 15°C, the AMI rate became significantly higher, demonstrating a linear association. This finding was compatible with the study results published previously that strong and significant association of higher rates of AMI hospitalization with cooler temperatures, particularly temperatures below 24°C, as evidenced in three Asian cities of Hong Kong, Kaohsiung and Taipei (14). Furthermore, a drop of 1° when the air temperature was below 15°C was associated with an increase of 1.6% of AMI incidence in our study. A similar result was found in a Portugal study, which concluded that a one-degree fall in physiological equivalent temperature during winter would lead to an increase of up to 2.2% of daily hospital admissions (15). Hence, colder weather results in a higher AMI rate.

In contrast, the relationship of AMI rate was found with hot weather in some studies. An increased risk of AMI hospitalization was associated with an increase in daily mean temperature was concluded in both Iranian and South Koran studies (16, 17). Additionally, another study revealed increased ST-segment elevation myocardial infarction (STEMI) occurrence in women ≤ 55 years was associated with higher temperature (18). Furthermore, extreme heat or hot spells were associated with AMI increase in a few studies (9, 19, 20). The specific weather condition was found to influence the onset of AMI and it was evidenced to be particularly associated with the maximum temperature 2 days before the onset of AMI only in summer in a Japanese study (21). However, such an association was not found in our study.

Due to regional and time constraints, the weather parameters obtained from the weather observation station could not be directly linked to the location of the patient when AMI occurred. Additionally, the time of the onset of AMI could be correctly defined in the NHIRD database. Furthermore, residents in need of emergency care in one region of Taiwan might be transported to another region because of resource disparities. For example, it was common for patients with AMI to be sent from the island parts or eastern parts of Taiwan to the northern or southern cities which were abundant in medical resources. Moreover, we considered subdividing Taiwan into seven geographic areas to identify distinct weather characteristics and influencing factors in this study. Two figures were supplemented to show at least four or six onsets of AMI from the northern to central areas of Taiwan between 2008 and 2011 (Supplementary Figures 1, 2). AMI continued to be more prevalent in the north than in the south. Due to the more minor residents in the northeast area, the results were still unchangeable as they merged into the northern region in Supplementary Figure 2. Importantly, the estimated values for statistically significant parameters varied little between these seven models. Hence, the observed values of the weather factors varied little when Taiwan was divided into seven geographic areas; therefore, we decided to look at the parameters as of the whole of Taiwan for our study.

The reason that a higher onset rate of AMI was associated with a lower maximum wind speed is undetermined. In Taiwan, a few typhoons approach frequently in summer and autumn, and a seasonal prevailing wind blows from the northeast in winter. Wind speed is influenced in these two specific kinds of weather; however, no significant correlation was found between the onset of AMI and these two specific kinds of weather in this study. Nevertheless, a study focusing on the influence of the changes in atmospheric state on AMI showed that a higher wind speed is associated with admission for AMI (22). Additionally, increased incidence of AMI was found correlated with the rapid increase in atmospheric pressure (23). Although mountains occupied more than 50% of Taiwan, more than 90% of citizens live on the ground. Therefore, atmosphere pressure was not analyzed in our study, however, patients who subsequently suffered from an AMI event may stay indoors. Further studies on the relationship of the onset of AMI with wind speed and atmospheric pressure are needed to clarify whether the onset of AMI is associated with the change of wind speed.

The onset of AMI was related to sunshine time when the GAM was used in the study, but sunshine time may be associated with minimum temperature. Both sunshine time and minimum temperature were statistically significant in GAM. Sunshine time was an independent factor in the onset of AMI. A multiethnic and multination epidemiological study revealed the circadian variation of STEMI onset. STEMI is markedly decreased in the summer season, and Vitamin D was correlated with the findings (24). In an observational study, patients with AMI had a very high prevalence of vitamin D deficiency (25). A lower onset rate of AMI was associated with increased sunshine time, indicating Vitamin D level might play an important role in the cascade of the onset of AMI and further studies are warranted.

When the medical histories of patients were reviewed to evaluate the onset rate AMI, age, male sex, heart failure, hypertension, diabetes mellitus, hyperlipidemia, COPD, and CKD were found to show significant association. A lowered onset rate of AMI was noticed to be related to the use of antiplatelet agents and statins. Interestingly, the results of univariate logistic regression (Table 2) showed that the use of statin is negatively related to the occurrence of AMI (OR = 0.009, *P* < 0.0001). On the contrary, the coefficient of statin use was significantly positively related to the daily onset rate of AMI when analyses were performed in the linear part of the GAM (*p* < 0.001; Table 4-1), whereas it was insignificant in the non-linear part of the GAM (*p* = 0.4854; Table 4-2). The medication compliance of statin use could not be obtained from the study database and on-treatment serum cholesterol level was not available in the claim database. Further studies are needed to investigate the causality of onset and statin use of AMI and how weather factors influence the causation. Besides, a lowered onset rate of AMI was associated with AF and MI. A multi-country study using the World Health Organization data showed that coronary events are more common in cold weather, but the excess is not greater in people with a history of heart disease (26). The risk of AMI was not increased in the patients with underlying heart diseases, except for heart failure. This might be attributed to the antiplatelet or anticoagulation therapies for the patients with prior MI or AF.

The novel GAM model we used for this study is, to our knowledge, the first model to predict the daily onset rate of AMI with meteorological factors. Models for AMI published previously were used to predict mortality (27–29), poor coronary collateral circulation (30), and readmission (31). By using our model of GAM, a precise onset rate of AMI is to be predicted in advance, allowing resources for emergency care such as medical manpower, equipment, facilities, and pharmaceutical supply to be allocated adequately to save lives. Furthermore, when meteorological factors are changed apparently to give rise to the estimated daily onset rate of AMI, a warning message might be sent to all citizens with high cardiovascular risks *via* an application software for mobile phones by the emergency medical technician team of the city government. People might thus become aware of AMI and alert for medical help when AMI symptoms or signs are noted, especially for those with high cardiovascular risks. The GAM model we made used the data collected between 2008 and 2011, however, some new antithrombotic agents had emerged in the recent decade, leading to the revision of the treatment guidelines for cardiovascular diseases. Besides, one topic becomes popular when people talk about warming the earth. One of the most visible consequences of a warming world is an increase in the intensity and frequency of extreme weather events. These factors might potentially influence the accuracy of our model, suggesting more recent data are used to improve its accuracy.

Our study has several limitations. First, no clinical data of the study patients such as blood pressure, heart rate, glycohemoglobin levels, and serum cholesterol levels could not be obtained in the NHIRD, and analyses of the factors then became impossible. The effect of statin therapy on serum cholesterol levels could not be estimated. Second, ICD coding biases might exist in the NHIRD because the symptoms of a disease might be interpreted differently to lead to a different diagnosis among doctors. Third, air pollution, PM2.5 for example, was not analyzed in this study. MI was found to be related to air pollution, which was evidenced in some studies published previously. Hence, the severity of air pollution might have some impact on the onset of AMI. Fourth, the persistence of medication was not investigated and the prescriptions of clopidogrel and statins might be underestimated because they are sometimes out-of-pocket items if the NHIA reimbursement is not allowed. Fifth, the weather in the northern and central region of Taiwan is subtropical and is it is tropical in the southern region. Therefore, the results and conclusion of this study should be carefully interpreted and applied to other geographical environments.

## Conclusion

Our study evidenced an increase in AMI occurrence in colder weather and presented a novel GAM model that predicts the daily onset rate of AMI and offers potential benefits to the public. The influence of wind speed on the onset of AMI remains uncertain.

## Data Availability Statement

The raw data supporting the conclusions of this article will be made available by the authors, without undue reservation.

## Author Contributions

C-IC agree to be accountable for all aspects of the work in ensuring that questions related to the accuracy or integrity of any part of the work are appropriately investigated and resolved and provide approval for publication of the content. C-YL and P-JW drafting the work or revising it critically for important intellectual content. All authors contributed to manuscript revision, read, and approved the submitted version.

## Funding

This research was supported by grants from Chang Gung Memorial Hospital (CGMRPG8B0012).

## Conflict of Interest

The authors declare that the research was conducted in the absence of any commercial or financial relationships that could be construed as a potential conflict of interest.

## Publisher's Note

All claims expressed in this article are solely those of the authors and do not necessarily represent those of their affiliated organizations, or those of the publisher, the editors and the reviewers. Any product that may be evaluated in this article, or claim that may be made by its manufacturer, is not guaranteed or endorsed by the publisher.
