# Supplementary figures and images for: Weather Impact on Acute Myocardial Infarction Hospital Admissions With a New Model for Prediction: A Nationwide Study
Source: Front Cardiovasc Med. 2021 Dec 14;8:725419. doi: 10.3389/fcvm.2021.725419 (PMC8712757; doi:10.3389/fcvm.2021.725419)

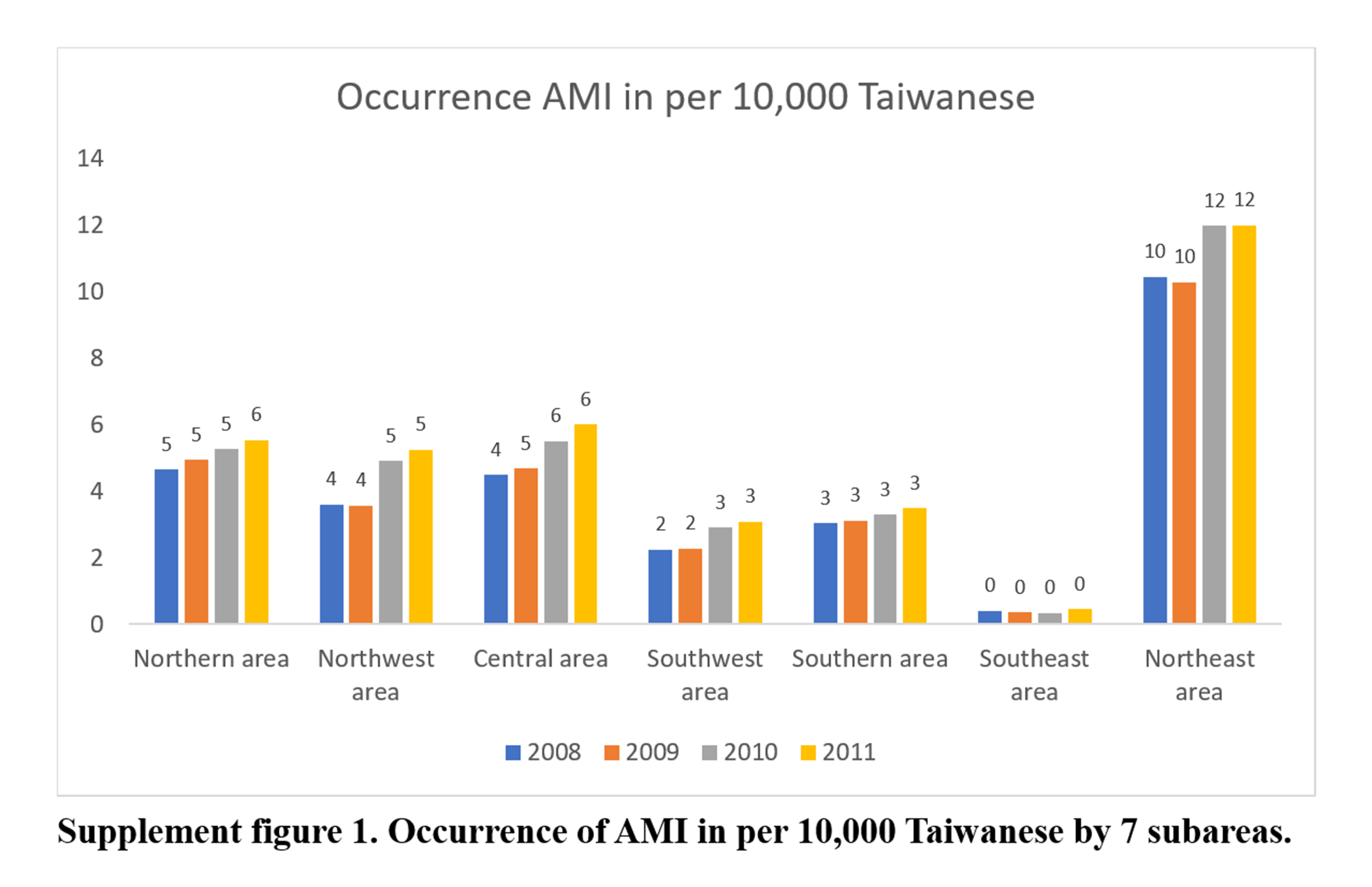

Supplement: Supplementary file 1 [file Image_1.TIF]

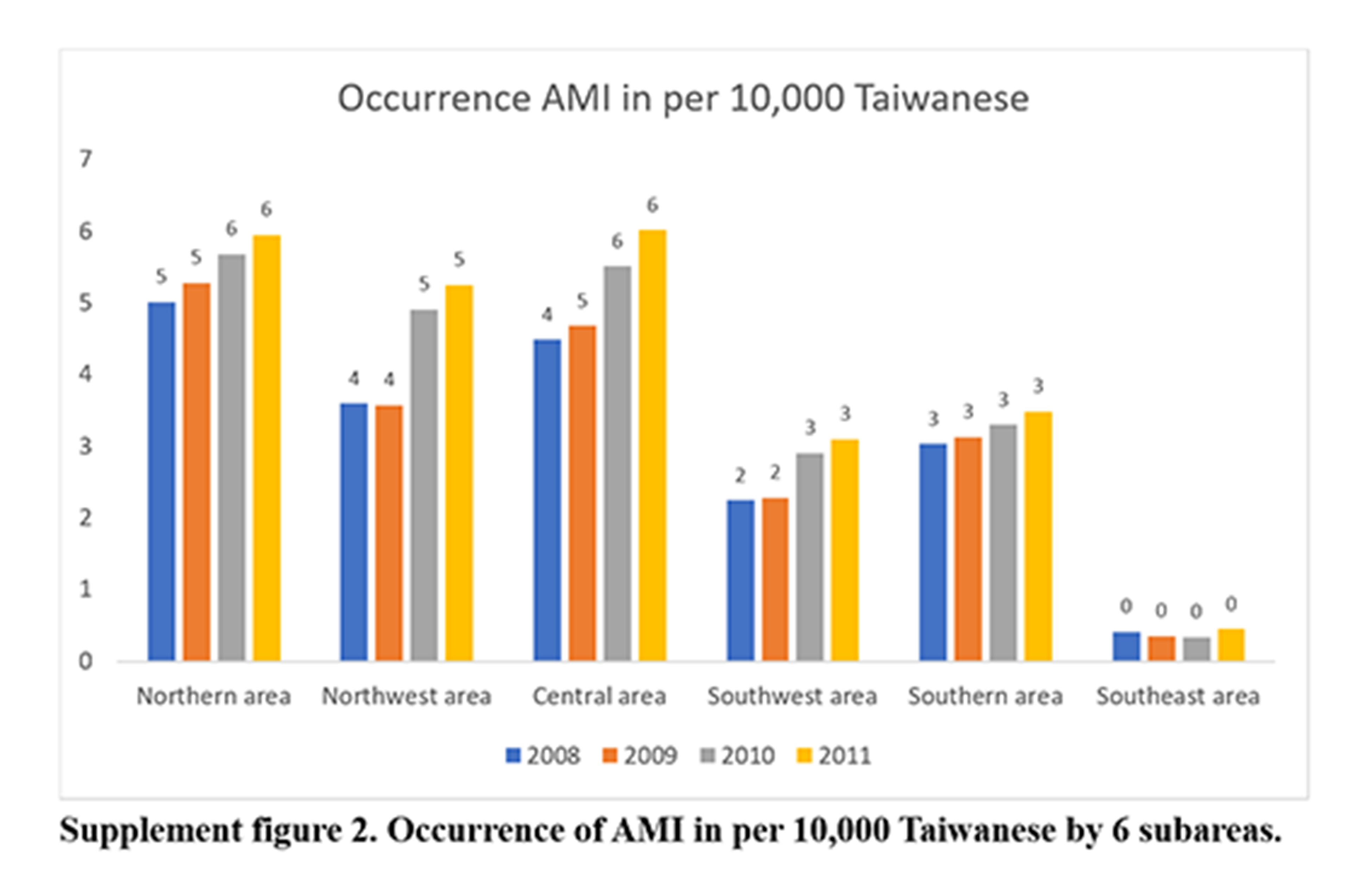

Supplement: Supplementary file 2 [file Image_2.TIF]
